# Norwalk-like calicivirus genes in farm animals.

**DOI:** 10.3201/eid0601.000106

**Published:** 2000

**Authors:** W H van Der Poel, J Vinjé, R van Der Heide, M I Herrera, A Vivo, M P Koopmans

**Affiliations:** National Institute of Public Health, Bilthoven, The Netherlands. Wim.van.der.Poel@rivm.nl

## Abstract

Viruses closely related to Norwalk-like viruses (NLVs) were recently found in stored stool samples from two calves (United Kingdom and Germany) and four pigs (Japan), sparking discussions about the potential for zoonotic transmission. To investigate if NLVs are commonly present in farm animals, pooled stool samples from 100 pig farms, 48 chicken farms, 43 dairy cow herds, and 75 veal calf farms from the Netherlands were assayed by reverse transcription- polymerase chain reaction amplification, using primers specific for the detection of NLVs from humans. NLV RNA was detected in 33 (44%) of the specimens from veal calf farms and two (2%) specimens from pig farms. Our data show that NLV infections until recently thought to be restricted to humans occur often in calves and sometimes in pigs. While zoonotic transmission has not been proven, these findings suggest that calves and pigs may be reservoir hosts of NLVs.


### Norwalk-Like Calicivirus Genes in Farm Animals

**Wim H.M. van der Poel,* Jan Vinjé,* Reina van der Heide,* Maria-Inmaculada Herrera,† Amparo Vivo,† and Marion P.G. Koopmans***

*National Institute of Public Health and the Environment, Bilthoven, the Netherlands; and †Instituto de Salud Carlos III, Majadahonda, Madrid, Spain

Viruses closely related to Norwalk-like viruses (NLVs) were recently found in stored stool samples from two calves (United Kingdom and Germany) and four pigs (Japan), sparking discussions about the potential for zoonotic transmission. To investigate if NLVs are commonly present in farm animals, pooled stool samples from 100 pig farms, 48 chicken farms, 43 dairy cow herds, and 75 veal calf farms from the Netherlands were assayed by reverse transcription-polymerase chain reaction amplification, using primers specific for the detection of NLVs from humans. NLV RNA was detected in 33 (44%) of the specimens from veal calf farms and two (2%) specimens from pig farms. Our data show that NLV infections—until recently thought to be restricted to humans—occur often in calves and sometimes in pigs. While zoonotic transmission has not been proven, these findings suggest that calves and pigs may be reservoir hosts of NLVs.

Caliciviruses infect animals and humans (1). Within the family *Caliciviridae*, four genera have been distinguished: vesivirus, lagovirus, Norwalk-like viruses (NLV), and Sapporo-like viruses (SLV) (2). The genera vesivirus and lagovirus contain a broad range of animal caliciviruses, but viruses in the NLV and SLV genera until recently have been found only in humans. In recent years NLVs, also known as small round-structured viruses, have emerged as a common cause of infectious gastroenteritis in all age groups and the main cause of outbreaks of gastroenteritis in restaurants and institutions such as nursing homes and hospitals (3-5).

Genetically, the human caliciviruses separate into at least three genetic clusters, called genogroups (GG), i.e., GGI and GGII NLV and the GGIII SLV (6). Each of these genogroups comprises genomically and antigenically diverse strains (7,8). This high degree of diversity results in at least 13 distinct genotypes for the NLVs (3,5,8-10). Many types of NLV cocirculate in the general population, causing sporadic cases and outbreaks. However, occasionally epidemics occur in which most outbreaks are caused by a single genotype (e.g., Lordsdale-like virus in the Netherlands in 1996) (5,11). Hypotheses for the mechanisms behind the emergence of epidemic types range from large-scale foodborne transmission of a single strain to introduction from a nonhuman reservoir. An indication for the latter was a recent report from Japan of the finding of NLV-like sequences in stool specimens from pigs (12). Enteric caliciviruses have also been detected in other animal species (calves, dogs, and chickens) (13-16). However, except for canine calicivirus (17), these viruses lack definitive sequence evidence linking them to the *Caliciviridae* (18). Molecular characterization of three calicivirus strains from cattle has recently been reported. The first virus (Tillamook virus; BCV-Bos1), described by Neill et al. (19), caused respiratory symptoms and was phylogenetically related to San Miguel sea lion virus and vesicular exanthema of swine virus, both within the genus vesivirus. Viruses in this genus have a broader host range (20). Two other viruses closely related to GGI NLVs (Jena virus 117/80 and Newbury agent type 2) were detected in feces from newborn calves with diarrhea (21,22). The

Address for correspondence: Wim H.M. van der Poel, Microbiological Laboratory for Health Protection, National Institute of Public Health and the Environment, Antonie van Leeuwenhoeklaan 9, 3720 BA Bilthoven, the Netherlands; fax: 31-30-274-4434; e-mail: Wim.van.der.Poel@rivm.nl.

similarity of the porcine and bovine sequences with those of NLVs found in humans suggests that a reservoir for these human pathogens may exist in farm animals.

We studied enteric caliciviruses in recently collected fecal samples from cattle, chicken, and swine in the Netherlands by using polymerase chain reaction (PCR) assays specific for NLVs found in humans. In addition, we analyzed (by sequence analysis) the genetic variation of detected calicivirus strains and compared it with that of prototype GGI and GGII NLV strains.

### Materials and Methods

### Fecal Specimens

Stool specimens from animals were collected at farms in the Netherlands as part of ongoing surveillance for potential zoonotic microorganisms associated with gastroenteritis in humans. From October 10, 1998, to April 21, 1999, samples were collected from 3- to 9-month-old fattening pigs at 100 pig farms of 22 to 1600 pigs. From January 1 to April 1, 1998, stool samples were collected from 75 veal calf farms of 38 to 930 calves and 43 dairy cattle herds of 25 to 180 cows. The age of the calves was 1 to 52 weeks (average 12 weeks), and of dairy cattle herds 4 to 6 years. From January 6 to April 21, 1998, samples were collected from ≤ 6-week-old chickens at 48 broiler farms, with 5,000 to 70,000 per farm.

### Sampling

The sampling strategy allowed monitoring for pathogens in a large number of animals and detection of microorganisms at farm level with a prevalence of 5% and 95% confidence (23). Fecal samples from calves, pigs, and chickens (20 to 60 per farm) were collected from animals housed in one randomly chosen farm building. Dairy cattle of one farm were regarded as a single herd, and stool samples were collected from randomly selected cows. Until tested, fecal samples were stored at -70°C in 15 g/L of Trypton Soya broth (TSB) (Oxoid CM 129) and 10% glycerol.

### Molecular Detection of NLVs by RT-PCR

For extraction of viral RNA, stool samples were resuspended in HBBS Hanks (Gibco BRL, Breda, Netherlands) to a final concentration of approximately 10%. These suspensions were centrifuged at 3,000 x g for 20 minutes, and 100 µl was used for RNA extraction by addition of a high-molarity solution of guanidinium isothiocyanate (GuSCN). Bound RNA was washed and eluted as previously described (24). To reduce the risk for contamination, specimens from different species were analyzed separately, and one negative control sample was included for every two stool specimens. A human stool sample positive for NLV by electron microscopy was included as positive control.

We used a single-round reverse transcription (RT)-PCR assay with a broadly reactive primer pair, which had been developed for the detection of NLVs in stool specimens from humans (5). For RT, 5 µl RNA was mixed with 4 µl 50 pmol JV13 primer. The solution was heated to 94°C for 2 minutes, cooled, and 6 µl RT buffer was added. The RT reaction was performed in a final volume of 15 µl, consisting of 10 mM Tris-HCl (pH 8.3), 50 mM KCl, 3 mM $MgCl_2$, 1 mM each of deoxynucleoside triphosphates (dNTPs), and 5 units of avian myeloblastoma virus RT (Boehringer Mannheim, Almere, Netherlands). The mixture was incubated for 1 hour at 42°C, heated for 5 minutes at 94°C to denature the enzyme, and then cooled. Five µl of the RT mixture was added to the PCR mix, containing 10 mM Tris-HCl (pH 9.2), 75 mM KCl, 1.5 mM $MgCl_2$, 0.2 mM dNTPs, 2.5 units AmpliTaq (Perkin Elmer, Nieuwerkerk a/d IJssel, Netherlands), and 15 pmol primer JV12. Mineral oil was added, and 40 amplification cycles, 1 minute at 94°C, 1.5 minute at 37°C, and 1 minute at 74°C each, were performed. The amplification products were analyzed by 2% agarose gel electrophoresis and visualized with UV after ethidium bromide staining.

For Southern blots, the RT-PCR products in the agarose gel were denatured by incubating in 0.5 M NaOH for 30 minutes and transferred to a positively charged nylon membrane (Boehringer, Almere, Netherlands) by vacuum blotting (Millipore, Etten-Leur, Netherlands). Hybridization of NLV RT-PCR products was performed as described previously (5).

### Sequence and Phylogenetic Analysis

The NLV RT-PCR products of expected size (326 bp) and 21 products of smaller size of the calf herd samples were excised from a 2% agarose gel and purified with a Qiaquick gel extraction kit (Qiagen, Hilden, Germany). Purified RT-PCR products were sequenced with the BigDye Terminator Cycle Sequencing Ready Reaction

Kit (Perkin Elmer Applied Biosystems, Foster City, CA) by using PCR primers. Nucleotide sequences were edited by using SeqEd (V1.03, Applied Biosystems), aligned by Geneworks (V2.5, Intelligenetics, Mountain View, CA), and imported into the Treecon software package (25). The confidence values of the internal nodes were calculated by performing 100 bootstrap analyses. Evolutionary trees for nucleotide sequences were drawn by using the Neighbor-joining method.

### Electron Microscopy

Electron microscopy was performed as recommended by Flewett (26) and Doane and Anderson (27), with model Philips 400T (Philips, Eindhoven, Netherlands) at 80 kV. Identification of virus particles was based on morphologic criteria, i.e., the size and characteristic surface morphology (28).

### Results

Twenty-five (33%) of 75 veal calf farm samples and 2 (2.0%) of 100 pig farm samples showed visible products of the expected size (326 bp). The gel electrophoresis and hybridization results are shown for eight strains (Figure 1). Hybridization with a set of probes used for detection of NLVs in humans yielded no positive reactions (Figure 1B); therefore, 46 products (25 of the expected size and 21 of a smaller size [Figure 1A, lane 7]) were sequenced. All 25 RT-PCR products of the expected size contained the GLPSG amino acid motif characteristic of viral RNA polymerases (29). The 21 products of smaller size revealed no viral sequences. The NLV-RT-PCR was negative for all pooled specimens from 43 dairy herds and 48 chicken farms.

By phylogenetic analysis, all calf calicivirus sequences formed a tight cluster closely related to NLVs from GGI with the highest similarity with the Newbury calf calicivirus (Figure 2) (22). Nucleotide sequence identities between the calf NLVs and GGI NLVs were 63% to 70% (75% to 77% amino acids). The swine virus sequences clustered as a separate lineage within GGII NLVs, with 69% to 71% nucleotide and 79% to 83% amino acid identity. In addition, the swine sequences strongly resemble (94% nucleotides, 100% amino acids) the swine calicivirus sequences from Japan. Partial polymerase sequences of Bo/NLV/176/1998/NET and Sw/NLV/34/1998/NET have been assigned GenBank accession numbers AF194183 and AF194184, respectively.

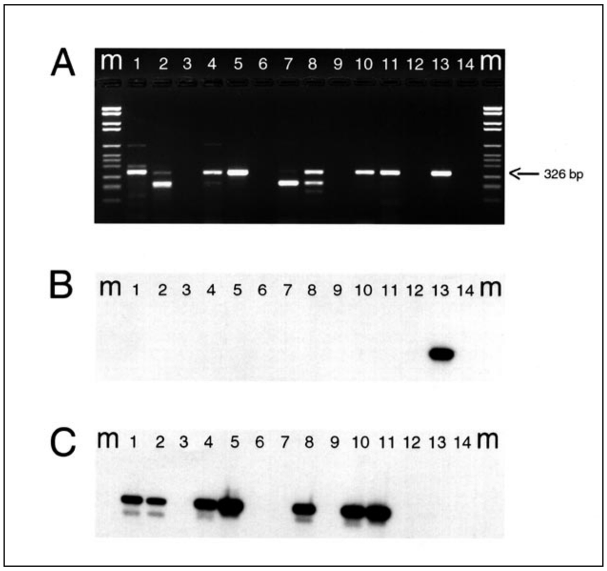

Figure 1. Results of ethidium bromide staining (panel A) and corresponding Southern hybridization (panels B and C) of RT-PCR products of eight calf herd (CH) samples (A) M= molecular mass marker, lane 1: CH124; lane 2: CH125; lane 3: water; lane 4: CH126; lane 5: CH138; lane 6: water; lane 7: CH139; lane 8: CH145; lane 9: water; lane 10: CH156; lane 11: CH176; lane 12: water; lane 13: human NLV positive control (5); lane 14: water. For Southern blot hybridizations, a set of probes used to detect NLVs in humans (B) and calves (C).

Since the calf and pig strains did not hybridize to the probe mixture for detection of NLVs from humans, specific probes were developed based on the consensus sequence of RT-PCR products of 10 calf herd samples and on the pig farm samples, respectively. The probe sequences were: RH1(calf) (5'-GGATGTGGTGCAGGCAA AC-3') and RH2(pig) (5'-TCCGCATCTCTATCGT GG-3'). Hybridizations with the calf probe (RH1) confirmed all RT-PCR-positive calf samples after 15 minutes exposure to an ECL hyperfilm (Figure 1C). Overnight exposure to ECL hyperfilm revealed eight more positives, for 33 (44%) positive calf farm samples.

All veal calf farm samples were screened by electron microscopy for viruses (Table). Particles with NLV morphologic features were found in only one specimen (Figure 3), in which the presence of calicivirus sequence was confirmed by RT-PCR and hybridization (Table).

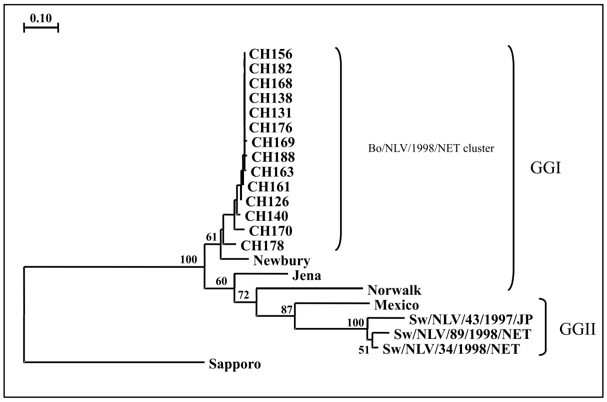

Figure 2. Phylogenetic relationships among human and animal NLVs clustering in genogroup (GG) I and II, based on a 145-bp nucleotide sequence within RNA polymerase gene. Dendrogram includes 14 calf herd (CH) sequences from the bovine NET/98 cluster (CH138, CH156, CH131, CH168, CH182, CH176 [Bo/NLV/176/1998/NET], CH163, CH188, CH169, CH161, CH126, CH140, CH170, and CH178 described in this study), and sequences from Norwalk virus (NV), Mexico virus (MX), Newbury agent (NA), Jena virus (JV), Sw/NLV/43/1997/JP, and the swine sequences described in this study: Sw/NLV/34/1998/NET and Sw/NLV/89/1998/NET. The SLV strain Sapporo (SAP) was included for comparison. Sequences of prototype strains of NLV were obtained from GenBank (Accession numbers: NV [M87661], MX [U22498], Newbury agent [AF097917], Jena virus [AJ011099], Sw/NLV/43/1997/JP [AB009412] and SAP [S77903]). Bootstrap values for each node are given if >50%.

Table. Viruses and virus particles detected by electron microscopy and corresponding Norwalk-like virus (NLV) RT-PCR results (including hybridization with a calf-NLV specific probe) in 16 of the 75 calf herd samples

| Sample | RT-PCR[a] results | EM results |
|---|---|---|
| CH121[b] | Neg | Adenovirus |
| CH127 | Neg | Parvovirus-like (21 nm)<br>Flavivirus-like particles (34-44 nm) |
| CH128 | Neg | Parvovirus-like (19 nm)<br>Rotavirus |
| CH141 | Neg | BDV-like[c] enveloped spherical particles (47 nm) |
| CH145 | Neg | Parvovirus-like (21 nm) |
| CH153 | Pos | Rotavirus |
| CH155 | Neg | Rotavirus |
| CH156 | Pos | Parvovirus-like (25-27 nm) |
| CH161 | Pos | Rotavirus |
| CH168 | Pos | Coronavirus-like particles |
| CH172 | Neg | Parvovirus-like (21 nm) |
| CH176 | Pos | SRSV[d] |
| CH178 | Pos | Rotavirus |
| CH185 | Pos | Rotavirus |
| CH186 | Neg | Rotavirus |
| CH187 | Neg | Coronavirus-like particles |

[a]RT-PCR = reverse transcription-polymerase chain reaction; [b]CH = calf herd; [c]BDV-like: bovine diarrhea viruslike.; [d]SRSV: small round-structured virus or NLV.

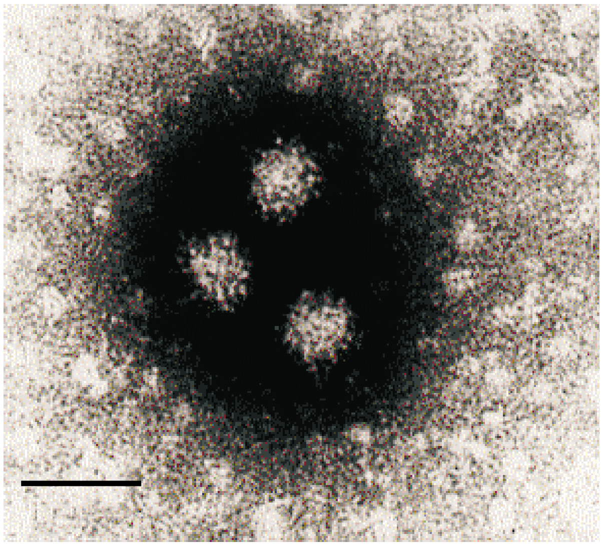

Figure 3. Electron microscopy showing NLV particles in a calf herd sample (CH176), negatively stained with 2% K-PTA, pH 7.0. Bar = 50nm.

### Conclusions

Until recently, humans were considered the sole host of NLVs. Recent studies in Japan and the United Kingdom, however, demonstrated calicivirus sequences in the caecum of pigs and in stored calf stool samples containing calicivirus-like particles by electron microscopy (12,21,22). Molecular characterization of calf enteric caliciviruses, named Newbury agent (22) and Jena virus (21), revealed that they were genetically related and more closely associated with GGI NLVs than with any other known calicivirus. In this study, we detected NLV nucleotide sequences in 33 (44%) of 75 of the pooled samples from veal calf farms.

Phylogenetic analysis of the detected sequences showed that they formed a tight cluster with the Newbury sequence (22) and were closely related to NLVs from GGI. Bridger et al. (30) demonstrated that calves can be infected with Newbury calicivirus. The detection of Newbury-like NLVs in pooled veal calf samples of 33 farms from different regions suggests that the species is a normal NLV host. Similarly, the close genetic relationship between calicivirus sequences detected in the pig samples in our study and those from Japan suggests that these viruses commonly infect pigs. The close similarity of porcine and bovine sequences to the NLVs infecting humans indicates the possibility of an animal reservoir for human infection.

However, these findings by no means prove zoonotic transmission. Genetically related viruses are commonly found in different species without resulting in apparent widespread interspecies transmission (e.g., rotavirus). On the other hand, zoonotic transmission cannot be excluded. The genetic distances between the animal and human NLVs are similar to the distances between the GGI and GGII strains, and epidemic spread of some NLV strains (5) led to the widespread—possibly global—emergence of a single predominant strain in the human population in 1995-96 (11). This epidemiologic observation resembles early descriptions of the vesicular exanthema of swine virus (VESV) epidemics (31). These viruses, belonging to another genus within the *Caliciviridae*, the vesiviruses, are known for their broad host range (20). The occasional epidemic spread of VESV was later linked to introduction of caliciviruses from an ocean reservoir (20). Therefore, interspecies transmission of NLVs is possible; the occasional widespread NLV epidemics caused by a single strain may result from introductions of new NLV strains from an animal reservoir.

Reynolds et al. reported electron microscopy detection of caliciviruses in calf diarrhea outbreaks (32), and the bovine caliciviruses Newbury agent (22) and Jena virus (21) were pathogenic for calves under experimental conditions and in field studies (33). In our study, we did not record disease or death on the surveyed farms and therefore cannot determine whether these caliciviruses were associated with disease. Despite repeated attempts, experimental infection of different animal species with the prototype Norwalk virus has not been successful, with the exception of infection in chimpanzees (3). Our findings may lead to the development of an animal model for the NLVs of humans; for example, a pig model would enable studies of (mucosal) immunity following NLV infection (1).

The low prevalence of Norwalk-like calicivirus in swine farms (2 per 100) is in agreement with the findings reported from Japan (12). Electron microscopy confirmed the calicivirus origin of the detected NLV sequences in only one calf sample. This low number of positives by electron microscopy was not surprising, given the greater sensitivity of RT-PCR and the use of pooled specimens for screening. NLVs in humans typically are shed at relatively low levels.

The absence of NLV sequences in all 43 dairy herd specimens may be explained by use of an inappropriate primer pair, which was optimized for detection of NLVs of humans (4) or by an acquired strain-specific immunity in older animals that prevents repeated infections. A third possibility is that levels of shedding in adult animals are very low, precluding virus detection in pooled specimens. The swine farm samples were all from fattening pigs at least 3 months old. We may have found a higher prevalence in recently weaned pigs, as the prevalence of other enteric pathogens is highest in young piglets (34).

Our findings raise important questions about the host range of NLVs. It is unclear if animal NLVs form genetically distinct stable lineages or are part of a common pool of viruses circulating between animals and humans. If calves or swine indeed are reservoirs, the prevalence of calicivirus should be determined in both healthy and diseased cattle and swine and methods to detect such interspecies transmissions at an early stage should be developed, in collaborative research by public health and veterinary sciences.

### Acknowledgments

We thank A.W. van de Giessen and ing. W.D.C. Deisz for volunteering fecal specimens from the monitoring study for zoonotic enteric pathogens, and A.M. Henken and A.M. de Roda Husman for critically reading the manuscript.

This research was financially supported and approved by the Dutch Inspectorate for Health Protection, Commodities and Veterinary Public Health.

Dr. van der Poel is a veterinary virologist in the Microbiological Laboratory for Health Protection, National Institute of Public Health and the Environment, the Netherlands. His research involves viral zoonoses and foodborne virus infections.

### References

1. Kapikian AZ, Estes MK, Chanock M. Norwalk group of viruses. In: Fields BN, Knipe DM, Howley PM, Channock RM, Melnick JL, Monath TP, et al., editors. Fields virology. 3rd ed. Vol. 1. Philadelphia (PA): Lippincott-Raven; 1996. p. 783-810.
2. Pringle CR. Virus taxonomy—San Diego 1998. Arch Virol 143:1449-59.
3. Green KY. The role of human caliciviruses in epidemic gastroenteritis. Arch Virol Suppl 1997;13:153-65.
4. Vinjé J, Koopmans MPG. Molecular detection and epidemiology of small round structured viruses in outbreaks of gastroenteritis in the Netherlands. J Infect Dis 1996;174:610-5.

5. Vinjé J, Altena SA, Koopmans MPG. The incidence and genetic variability of small round-structured viruses in outbreaks of gastroenteritis in the Netherlands. J Infect Dis 1997;176:1374-8.
6. Matson DO, Zhong WM, Nakata S, Numata K, Jiang X, Pickering LK, et al. Molecular characterization of a human calicivirus with sequence relationships closer to animal caliciviruses. J Med Virol 1995;45:215-22.
7. Ando T, Mulders MN, Lewis DC, Estes MK, Monroe SS, Glass RI. Comparison of the polymerase region of small round-structured virus strains previously classified in three serotypes by solid-phase immune electron microscopy. Arch Virol 1994;135:217-26.
8. Jiang X, Cubitt WD, Berke T, Zhong W, Dai X, Nakata S, et al. Sapporo-like human caliciviruses are genetically and antigenically diverse. Arch Virol 1997;142:1813-27.
9. Green J, Vinjé J, Gallimore C, Koopmans MPG, Hale A, Brown D. Capsid protein diversity among Norwalk-like caliciviruses In press 2000.
10. Vinjé J, Deijl H, van de Heide R, Lewis D, Hedlund K-O, Svensson L, et al. Molecular detection and epidemiology of Sapporo-like viruses. J Clin Microbiol. In press 2000.
11. Noel JS, Fankhauser RL, Ando T, Monroe SS, Glass RI. Identification of a distinct common strain of Norwalk-like viruses having a global distribution. J Infect Dis 1999;179:1334-44.
12. Sugieda M, Nagaoka H, Kakishima Y, Ohshita T, Nakamura S, Nakajima S. Detection of Norwalk-like virus genes in the caecum contents of pigs. Arch Virol 1998;143:1215-21.
13. Granzow H, Schirmeier H. Identification of 32 nm viruses in faeces of diarrhoeic calves by electron microscopy. Monatshafte Veterinärmedizin 1985;40:228-9.
14. Herbst W, Lange H, Krauss H. Elektronenmikroskopischer Nachweis von calicivirus-ähnlichen Partikeln im Kot durchfallkranker Kälber. Deutsche Tierärztliche Wochenschrift 1987;94:381-440.
15. Mochizuki M, Kawanishi A, Sakamoto S, Tashiro S, Fujimoto R, Ohwaki M. A calicivirus isolated from a dog with fatal diarrhoea. Vet Rec 1993;132:221-2.
16. Reckling KF. Use of electron microscopy for observation of small round viruses in faecal samples collected from calves and animals with diarrhoea. Monatshafte Veterinärmedizin 1987;42:272-5.
17. Hashimoto M, Rierink F, Tohya Y, Mochizuki M. Genetic analysis of the RNA polymerase gene of caliciviruses from dogs and cats. J Vet Med Sci 1999;61:603-8.
18. Cubitt D, Bradley MJ, Carter S, Chiba S, Estes MK, Saif LJ, et al. Viral taxonomy, classification and nomenclature; sixth report of the committee on the taxonomy of viruses. Arch Virol Suppl 1997;ESVV 77-82, Reading, United Kingdom.
19. Neill JD, Meyer RF, Seal BS. Genetic relatedness of the caliciviruses: San Miguel sea lion and vesicular exanthema of swine viruses constitute a single genotype within the *Caliciviridae*. J Virol 1995;69:4484-8.
20. Smith AW, Skilling DE, Cherry N, Mead JH, Matson DO. Calicivirus emergence from ocean reservoirs: zoonotic and interspecies movements. Emerg Infect Dis 1998;4:13-20.
21. Liu BL, Lambden PR, Gunther H, Otto P, Elscher M, Clarke IN. Molecular characterization of a bovine enteric calicivirus: relationship to the Norwalk-like viruses. J Virol 1999;73:819-25.
22. Dastjerdi AM, Green J, Gallimore CI, Brown DWG, Bridger J. The bovine newbury agent-2 is genetically more closely related to human SRSVs than to animal caliciviruses. Virology 1999;254:1-5.
23. Giessen van de AW, Frankena K, Leeuwen van WJ, Notermans SHW. An approach for monitoring salmonella serotypes in farm animals. Proceedings of the Symposium "Salmonella and salmonellosis." Ploufragan 1992:375-85.
24. Boom R, Sol CJA, Salimans MMM, Jansen CL, Wertheim-van Dillen, van der Noordaa J. Rapid and simple method for purification of nucleic acids. J Clin Microbiol 1990;28:495-503.
25. Van der Peer Y, De Wachter R. TREECON for windows: a software package for the construction and drawing of evolutionary trees for the Microsoft Windows environment. Computer Applications in Bioscience 1994;10:569-70.
26. Flewett TH. Electron microscopy in the diagnosis of infectious diarrhea. J Am Vet Med Assoc 1978;173:538-43.
27. Doane FW, Anderson N. Pretreatment of clinical specimens and viral isolates. In: Electron microscopy in diagnostic virology. Cambridge: Cambridge University Press; 1987. p. 4-10.
28. Caul EO, Appleton H. The electron microscopical and physical characteristics of small round human fecal viruses: an interim scheme for classification. J Med Virol 1982;9:257-65.
29. Bruenn JA. Relationships among the positive strand and double-strand RNA viruses as viewed through their RNA-dependent RNA polymerases. Nucleic Acids Res 1991;25:217-26.
30. Bridger JC, Hall GA, Brown JF. Characterization of a calici-like virus (Newbury agent) found in association with astrovirus in bovine diarrhea. Infect Immun 1984;43:133-8.
31. Smith AW, Akers TG. Vesicular exanthema of swine. J Am Vet Med Assoc 1976;169:700-3.
32. Reynolds DJ, Morgan JH, Chanter N, Jones PW, Bridger JC, Debney TG, et al. Microbiology of calf diarrhoea in southern Britain. Vet Rec 1986;119:34-9.
33. Günther H, Otto P, Heilman P. Studies into diarrhoea of young calves. Sixth communication: detection and determination of pathogenicity of a bovine corona virus and an undefined icosahedric virus. Archiven Experimenteller Veterinärmedizin (Leipzeig) 1984;38:781-92.
34. Will LA, Paul PS, Proescholdt TA, Aktar SN, Flaming KP, Janke BH, et al. Evaluation of rotavirus infection and diarrhea in Iowa commercial pigs based on epidemiologic study of a population represented by diagnostic laboratory cases. J Vet Diagn Invest 1994;6:416-22.
